# Neurological Complications after Neonatal Bacteremia: The Clinical Characteristics, Risk Factors, and Outcomes

**DOI:** 10.1371/journal.pone.0105294

**Published:** 2014-11-03

**Authors:** Shih-Ming Chu, Jen-Fu Hsu, Chiang-Wen Lee, Reyin Lien, Hsuan-Rong Huang, Ming-Chou Chiang, Ren-Huei Fu, Ming-Horng Tsai

**Affiliations:** 1 Division of Pediatric Neonatology, Department of Pediatrics, Chang Gung Memorial Hospital, Taoyuan, Taiwan; 2 Division of Neonatology and Pediatric Hematology/Oncology, Department of Pediatrics, Chang Gung Memorial Hospital, Yunlin, Taiwan; 3 College of Medicine, Chang Gung University, Taoyuan, Taiwan; 4 Department of Nursing, Division of Basic Medical Sciences, and Chronic Diseases and Health Promotion Research Center, Chang Gung University of Science and Technology, Chia-Yi, Taiwan; 5 Research Center for Industry of Human Ecology, Chang Gung University of Science and Technology, Taoyuan, Taiwan; University of Iowa Carver College of Medicine, United States of America

## Abstract

**Background:**

Neonates with bacteremia are at risk of neurologic complications. Relevant information warrants further elucidation.

**Study Design:**

This was a retrospective cohort study of neonates with bacteremia-related neurologic complications (BNCs) in a tertiary-level neonatal intensive care unit (NICU). A systemic chart review was performed conducted to identify clinical characteristics and outcomes. A cohort of related conditions was constructed as the control group. Logistic regression analysis was used to identify independent risk factors for BNC.

**Results:**

Of 1037 bacteremia episodes, 36 (3.5%) had BNCs. Twenty-four cases of BNCs were related to meningitis, five were presumed meningitis, and seven occurred after septic shock. The most common causative pathogens were Group B streptococcus (41.7%) and *E. coli* (16.7%). The major BNCs consisted of seizures (28), hydrocephalus (20), encephalomalacia (11), cerebral infarction (7), subdural empyema (6), ventriculitis (8), and abscess (4). Eight (22.8%) neonates died and six (16.7%) were discharged in critical condition when the family withdrew life-sustaining treatment. Among the 22 survivors, eight had neurologic sequelae upon discharge. After multivariate logistic regression analysis, neonates with meningitis caused by Group B streptococcus (adjusted odds ratio [OR]: 8.90, 95% confidence interval [CI]: 2.20–36.08; *p* = 0.002) and combined meningitis and septic shock (OR, 5.94; 95% CI: 1.53–23.15; *p* = 0.010) were independently associated with BNCs.

**Conclusions:**

Neonates with bacteremia-related neurologic complications are associated with adverse outcomes or sequelae. Better strategies aimed at early detection and reducing the emergence of neurologic complications and aggressive treatment of Group B streptococcus sepsis are needed in neonates with meningitis and septic shock.

## Introduction

Neonatal bacteremia remains one of the major infectious problems in the neonatal intensive care unit (NICU) [1], [2], with mortality rates of 10%–30% [3]–[5]. It is also associated with increased medical costs, prolonged hospital stay and potentially poor long-term neurodevelopmental outcomes [6]–[8]. Neonatal bacterial meningitis is a severe infectious disease that often occurs concomitantly with the onset of bacteremia, especially those caused by Group B streptococcus (GBS), *E. coli* or other Gram-negative pathogens [9]–[11]. Neonatal meningitis has been associated with a variety of neurologic complications including seizure, hydrocephalus, arachnoiditis, subdural empyema, and encephalopathy [12]–[15].

Neurological complications after neonatal bacteremia with or without meningitis are important, because they can be life-threatening and may require neurosurgical treatment. There are a large number of studies on the incidence, risk factors, microbiology, and mortality of neonatal bacteremia or meningitis [1]–[8], but there is paucity of literature regarding acute and subacute neurological morbidities caused by neonatal bacteremia. Moreover, an episode of bacteremia presenting with severe sepsis or septic shock may be associated with neurological sequelae, probably due to brain hypoperfusion, transient hypoxia, or metabolic acidosis [16]. Prompt identification and aggressive intervention for these neurological complications will result in a more favorable outcome. In this study, we examined the occurrence, characteristics, risk factors and treatment for acute neurological complications after bacteremia in neonates.

## Materials and Methods

### Study design, setting, and patients

A database search of all positive blood culture-proven neonatal bloodstream infections at the NICU of Chang Gung Memorial Hospital (CGMH) between January 2004 and December 2011 was performed. The NICU is an academic, tertiary-level medical center with a total of 49 beds equipped with mechanical ventilator and 28 beds with special care nurseries. Bacteremia-associated neurological complication (BNC) was defined as any newly neurological symptoms or signs and abnormalities on neuroimaging study (Transcranial ultrasound, computed tomography [CT] scan or magnetic resonance imaging [MRI]) that occurred soon after an episode of bacteremia, or judged by a clinical neonatologist to be directly resulted from an episode of bacteremia. Fulminant episodes of neonatal bacteremia with early mortality within 48 hours from onset were not enrolled. This study was approved by the institutional review board of CGMH, with a waiver of informed consent. However, all patient records/information was anonymized and de-identified prior to analysis.

### Case finding

The hospital records of all neonates with bloodstream infection were reviewed for evidence of neurological complications. Patients were selected to undergo detailed chart review when: 1) a lumbar puncture or neuroimaging study was performed; 2) a neurologic consultation was arranged; 3) a seizure was documented in the discharge or progression note; 4) a neurologic surgery, such as ventriculoperitoneal shunting or extraventricular drainage, was performed; or 5) experience of severe sepsis or septic shock during hospital course.

### Study definitions

#### Neonatal bacteremia (or bloodstream infection)

Neonatal bacteremia was defined as according to the criteria from the Centers for Disease Control (CDC) and Prevention [17]. Early-onset sepsis and late-onset sepsis were defined as the presence of clinical sepsis and at least one positive blood culture obtained before and after the first 72 hours of life, respectively [6]–[8]. Blood cultures positive for organisms generally considered as contaminants, including corynebacterium, propionibacterium, penicillium, and diphtheroids, were excluded from analysis. Records of patients with blood culture positive for CoNS were reviewed, and the CDC criteria for CoNS bacteremia were strictly applied [17].

#### Chronic medical conditions

All comorbidities of prematurity, including respiratory distress syndrome (RDS), intraventricular hemorrhage (IVH), bronchopulmonary dysplasia (BPD), and periventricular leukomalacia (PVL) were based on the latest updated diagnostic criteria in the standard textbook of neonatology [18]. Congenital anomalies in this study included all neonates with either documented or undocumented syndrome, chromosome abnormalities, and genetic or metabolic disorders, but not simple cleft palate or polydactyly.

Bacteremia-related neurological complications included:

1. Seizure: neonates without an underlying seizure disorder, brain pathology, or significant metabolic disturbance who had a repeated seizure attack or an abnormal epileptiform discharges on the electroencephalography after bacteremia that required regular anticonvulsants medications.
2. Post-infectious encephalopathy: Neonates who had consciousness change after stabilization of vital signs that lasted >24 hours after the onset of bacteremia.
3. Hydrocephalus and/or ventriculomegaly: documented by transcranial ultrasound after the onset of bacteremia, and in neonates without previous brain pathology.
4. The presence of any newly focal infections, including subdural empyema, arachnoiditis, ventriculitis, and spinal abscess or brain abscess.
5. Other neurologic complications: included neonates with encephalomalacia or cerebral infarction due to hypotension.

### Data collection

This neonatal database was fed by the neonatologist specialist every weekday since 2003 and contained information on basic demographic characteristics, records of all complications of prematurity and all nosocomial infections, summary of hospital courses, and final diagnosis of all patients. For all enrolled cases and controls, detailed data were retrieved by a systemic review of the medical record by using a structured data collection form. Two independent investigators (Dr. H.-R.H. and Dr. J.-F.H.) searched for the following variables: 1) presence and duration of altered mental status; 2) seizure activity along the entire hospital courses, or during or within 48 hours after onset of bacteremia; 3) results of cerebrospinal fluid analysis and findings of neuroimaging studies; and 4) antimicrobial regimens, treatment and hospital course, and clinical outcomes. In patients with a BNC, it was confirmed by a pediatric infectious disease specialist and a pediatric neurologist.

### Enrollment of the control group

All neonatal bacteremia, including early-onset sepsis and late-onset sepsis, were retrospectively reviewed to identify the characteristics that predisposed neonates at risk of developing BNCs. After identifying the subgroups vulnerable to have BNCs, those without BNCs after bacteremia were enrolled as the controls. All demographic data, clinical presentations, treatment and outcomes were compared between cases (neonates with BNC) and the controls.

### Statistical analysis

Tests of significance between means and proportions were carried out using either χ^2^ or Student *t* test, respectively. All *p* values were two tailed, and were considered to be statistically significant if the value was <0.05. Categorical data was tested for odds ratios (ORs). Unadjusted and adjusted OR and corresponding 95% confidence intervals (CIs) were derived to examine the risk factors for the development of BNC. We performed a multivariate analysis using a logistic regression model to examine the interaction among gestational age, which was defined as a categorical variable, and meningitis and septic shock. Only variables with a p value<0.1 will be enrolled into the final multivariate logistic regression model. All statistical calculations were performed using SPSS software version 15.0 (SPSS, Chicago, IL).

## Results

During the study period, 1037 episodes of neonatal bacteremia and 57 episodes of meningitis were identified in 769 patients in our NICU. After onset of bacteremia, neurological complications were identified in 36 (3.5%) patients (episodes) by brain sonography (n = 36) and/or CT scan (n = 36) and/or brain MRI (n = 33) and/or lumbar puncture (n = 31). When compared with neonatal bacteremia without neurological complications (Table 1), BNCs had a high percentage (23/36, 63.9%) of occurring in the late-preterm (gestational age 33–36 weeks) or term-born infants, and had an earlier onset of bacteremia, although the rate of early-onset sepsis was comparable. The causative pathogens in the neonatal bacteremia with BNCs were also significantly different from those without BNCs (Table 1). Furthermore, patients with bacteremia and BNCs were usually associated with more severe clinical manifestations and significantly higher severity of illness (judged by NTISS scores) [19] (Table 1).

**Table 1:** Clinical and laboratory features in neonates with bacteremia-associated neurological complications among 1037 episodes of bacteremia in the neonatal intensive care unit.

Characteristics	Bacteremia episodeswith neurologicalcomplications (n = 36)	Bacteremia episodeswithout neurologicalcomplications (n = 1001)	*P* value
Birth body weight (g), median (IQR)	2520.0 (1535.0–3017.5)	1340.0 (941.5–2032.5)*	<0.001
Gestational age (weeks), median (IQR)	36.0 (29.3–38.8)	30.0 (27.0–35.0)*	<0.001
Age at onset of bacteremia (days), median (IQR)	20.5 (6.0–30.3)	25.0 (14.0–50.0)	0.008
Early-onset sepsis,& n (%)	5 (13.9)	80 (8.0)	0.209
Late-onset sepsis,& n (%)	31 (86.1)	921 (92.0)	
Causative Pathogens, n (%)			<0.001
Group B Streptococcus	15 (41.7)	33 (3.3)	
Escherichia coli	6 (16.7)	79 (7.9)	
Other Gram-positive pathogens	5 (13.9)	544 (54.3)	
Other Gram-negative pathogens	10 (27.8)	248 (24.8)	
Fungus	0 (0)	52 (5.2)	
Polymicrobial pathogens	0 (0)	45 (4.5)	
Clinical manifestations, n (%)			
Septic shock	18 (50.0)	166 (16.6)	<0.001
Coagulopathy or gastrointestinal bleeding	20 (58.3)	242 (24.2)	<0.001
Disseminated intravascular coagulopathy	12 (33.3)	89 (8.9)	<0.001
Respiratory distress¶	27 (75.0)	605 (60.5)	0.084
Requirement of blood transfusion#	24 (66.7)	374 (37.4)	0.001
Concurrent meningitis	24 (66.7)	33 (3.3)	<0.001
NTISS score at most severe dayof bacteremia, mean±standard deviation	18.9±6.13	16.7±4.92	0.006
Outcomes, n (%)			
Sepsis attributable mortality	14/36 (38.9)	68/1001 (6.8)	<0.001
Overall mortality	14/36 (38.9)	90/769 (11.7)*	<0.001

*Data are 713 unique patients with late onset sepsis and 56 unique patients with early-onset sepsis.

¶ Indicating those required mechanical ventilators, including intubation or continuous positive airway pressure, during the treatment courses of bacteremia.

& Early-onset sepsis and late-onset sepsis are defined as clinical sepsis with positive blood culture obtained before and after first 72 hours of life, respectively.

# Indicating requirement of blood transfusions of red blood cell, platelet, or fresh frozen plasma.

IQR: interquartile range, NTISS: Neonatal Therapeutic Intervention Scoring System^19^.

The basic demographics of neonates with BNCs are summarized in Table 2. In these patients with BNCs, 24 patients (66.7%) had meningitis, five (13.9%) had negative CSF cultures but at least one individual CSF marker of meningitis, and seven (19.4%) had neurological sequelae after septic shock or severe sepsis. The BNCs included seizure (n = 28, 77.8%), ventriculomegaly (n = 26, 72.2%), hydrocephalus (n = 20, 55.6%), encephalomalacia (n = 11, 30.6%), cerebral infarction (n = 7, 19.4%), subdural empyema (n = 6, 16.7%), abscess (n = 4, 11.1%), ventriculitis (n = 8, 22.2%) and subdural effusion (n = 11, 30.6%).

**Table 2:** Pathogens, clinical manifestations, and demographics of neonates with bacteremia-associated neurological complications.

Characteristics	Total cases(n = 36)	Meningitis(n = 24)	PresumedMeningitis(n = 5)	Septic shock(n = 7)
Birth weight (g), median (IQR)	2520.0 (1535.0–3017.5)	2700.0 (2236.0–3127.5)	2100.0 (1360.0–2679.5)	1125.0 (800–2330.0)
Gestational age (weeks), median (IQR)	36.0 (29.3–38.8)	37.5 (33.5–39.8)	34.0 (29.0–37.5)	29.0 (25.0–36.0)
Gender (male/female), n (%)	19 (52.8)/17 (47.2)	15 (62.5)/9 (37.5)	2 (40.0)/3 (60.0)	2 (28.6)/5 (71.4)
Age of bacteremia onset (days), median (IQR)	20.5 (6.0–30.3)	21.5 (6.0–33.3)	10.0 (5.5–21.5)	21.0 (4.0–31.0)
Pathogens, n (%)				
Group B *Streptococcus*	15 (41.7)	14 (58.3)	1 (20.0)	0 (0)
* Escherichia coli*	6 (16.7)	3 (12.5)	3 (60.0)	0 (0)
Other Gram-positive pathogens	5 (13.9)	2 (8.3)	0 (0)	3 (42.9)
Other Gram-negative pathogens	10 (27.8)	5 (20.8)	1 (20.0)	4 (57.1)
Neurological complications after bacteremia				
Seizure	28 (77.8)	19 (79.2)	4 (80.0)	5 (71.4)
Ventriculomegaly/Hydrocephalus	26 (72.2)/20 (55.6)	17 (70.8)/12 (50.0)	4 (80.0)/4 (80.0)	5 (71.4)/4 (57.1)
Increased intracranial pressure (IICP)	26 (72.2)	17 (70.8)	4 (80.0)	5 (71.4)
Subdural effusion	11 (30.6)	8 (33.3)	1 (20.0)	2 (28.6)
Encephalomalacia	11 (30.6)	7 (29.1)	2 (40.0)	2 (28.6)
Ventriculitis	8 (22.2)	5 (20.8)	2 (40.0)	1 (14.3)
Subdural empyema	6 (16.7)	6 (25.0)	0 (0)	0 (0)
Abscess#	4 (11.1)	4 (16.7)	0 (0)	0 (0)
Cerebral infarction	7 (19.4)	4 (16.7)	1 (20.0)	2 (28.6)
Intracranial hemorrhage¶	7 (19.4)	4 (16.7)	1 (20.0)	2 (28.6)
Periventricular leukomalacia	4 (11.1)	2 (8.3)	0 (0)	2 (28.6)
Condition at discharge, n (%)				
Survival	22 (61.1)	17 (70.8)	2 (40.0)	3 (42.9)
Discharge without neurological sequelae	14 (38.9)	10 (41.7)	2 (40.0)	2 (28.6)
Discharge with neurological sequelae	8 (22.2)	7 (29.2)	0 (0)	1 (14.3)
Mortality	8 (22.2)	4 (16.7)	2 (40.0)	2 (28.6)
Withdraw life-sustaining treatment	6 (16.7)	3 (12.5)	1 (20.0)	2 (28.6)

# Including subdural abscess (2), and spinal cord abscess (2), and brain abscess (1).

¶ Including intraventricular hemorrhage (3), subependymal hemorrhage (3), subarachnoid hemorrhage (1), and hemorrhage on bilateral globus pallidus (1).

IQR: interquartile range.

The most common causative pathogens were GBS (15/36, 41.7%) and *E. coli* (6/36, 16.7%). Among neonates with meningitis, the most common pathogen was GBS (14, 58.3%). Other pathogens included *E. coli* (3), *K. pneumoniae* (1), *Chryseobacterium meningosepticum* (1), *L. monocytogenes* (1), Salmonella group D (1), *P. aeruginosa* (1), *E. cloacae* (1), and coagulase negative Staphylococcus (1). All of the meningitis events were concomitant with the onset of bacteremia, except for one case of Salmonella group D meningitis that occurred at one week after onset of bacteremia. In these patients, pre-existing neurological comorbidities were noted in only five patients, including IVH Gr II (2), IVH Gr III (2), and one patient had IVH Gr IV and hypoxic ischemic encephalopathy due to perianal asphyxia.

**Figure 1:**
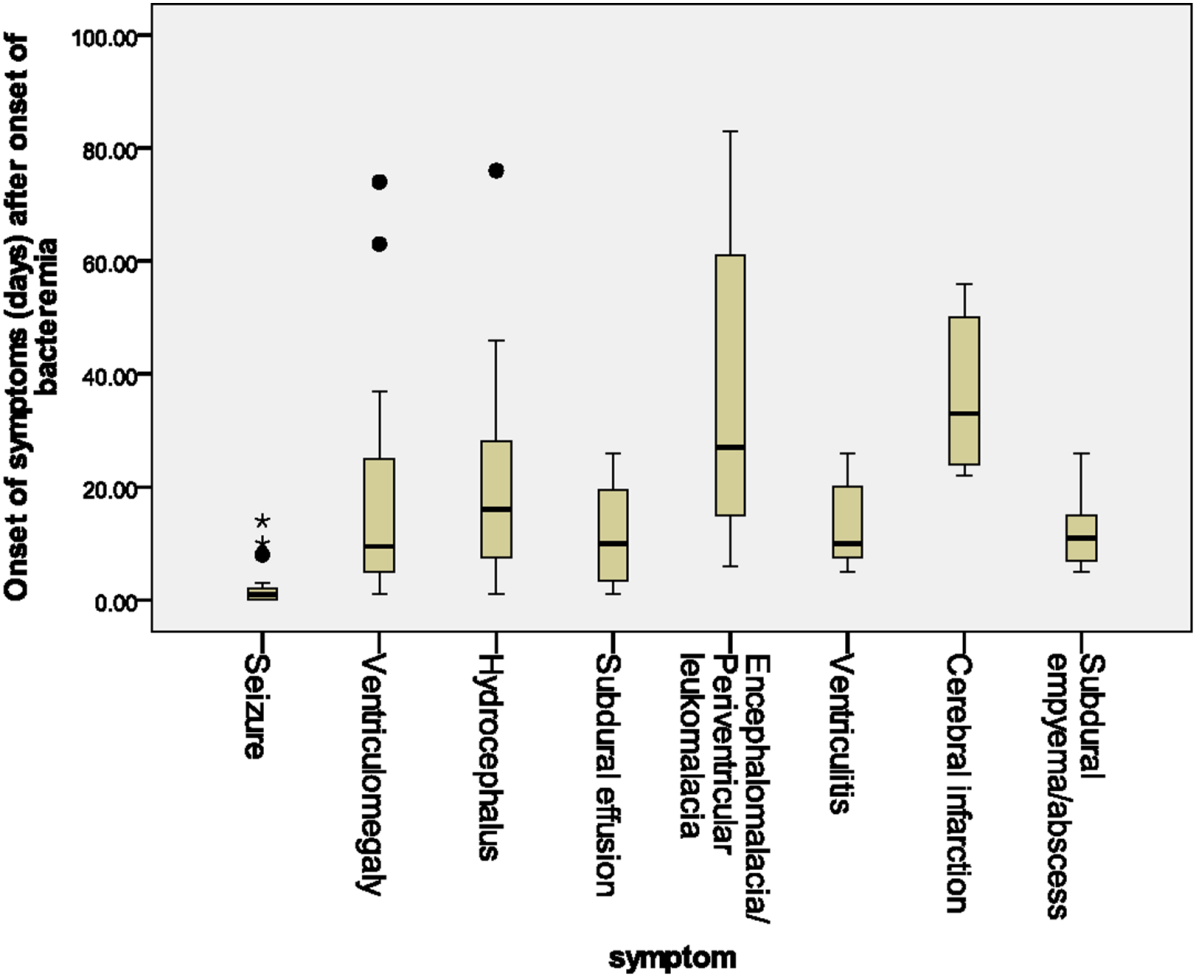
Time to diagnosis of various bacteremia-related neurological complications in the neonatal intensive care unit. Bacteremia onset was defined as when the blood culture sampling was obtained, whereas onset of neurological complication was defined at the symptom presentation or diagnosis by neuroimaging studies.

The time to diagnosis of all BNCs from onset of bacteremia with and without meningitis until BNCs is summarized in Figure 1. The median time from the onset of sepsis to neurologic symptoms was 10 days (range, 0–82 days, interquartile range [IQR], 3–24 days), with different symptoms highly correlated with their onsets. Seizure accounted for the earliest onset of neurological symptoms (median time from sepsis, 1 days, range: 0–14 days), and encephalomalacia and cerebral infarction were often the last to be detected (median time from onset of sepsis, 27 days, IQR, 20–56 days). The clinical features during this period included seizure (77.8%), feeding intolerance (86.1%), fever (11.1%), respiratory failure (19.4%), increased intracranial pressure (72.2%), bulging anterior fontanel (27.8%), and lethargy (22.2%). Among the 28 neonates with seizure, six patients had seizure as the initial symptom of bacteremia and seven (25%) were poorly controlled despite the administration of antiepileptic drugs for more than 48 hours. Nine patients had electrolyte imbalance, three patients developed central diabetes insipidus, and one had syndrome of inappropriate antidiuretic hormone secretion (SIADH).

Initial antibiotics that covered both Gram-positive and Gram-negative organisms were used for all thirty-six patients. Only two cases (pathogens: *C. meningosepticum* and *P. aeruginosa*) did not receive appropriate antibiotics within 24 hours after bacteremia onset. They were treated with penicillin, one of third generation cephalosporin (cefotaxime or ceftazidime), or carbapenem, based on the results and antimicrobial susceptibility patterns of blood and CSF cultures. The mean duration of antimicrobial therapy was 23.2±11.8 days (range, 7 to 38 days). Eighteen (50%) patients received neurosurgical treatment, including extraventricular drainage tube placement (n = 9), ventriculoperitoneal shunt (n = 9), subdural drainage (n = 3) and subdural-peritoneal shunt (n = 2). Four patients required multiple neurosurgical interventions.

Focal infectious complications occurred in fifteen patients, including seven had ventriculitis, four had subdural empyema, two had subdural abscess, one had subdural empyema plus brain abscess, and the last one had subdural empyema plus spinal cord abscess and arachnoiditis. All of these BNCs occurred after meningitis (12 patients with the following causative pathogens: GBS [6], *E. coli* [3], Salmonella group D [1], *P. aeruginosa* [1], and *C. meningosepticum* [1]) or presumed meningitis (3 cases). These BNCs were found at 4–22 days (median: 7 days) after the onset of bacteremia. Cranial MRI was performed in all of these patients and included diffusion-weighted imaging (DWI) hyperintense and apparent diffusion coefficient (ADC) hypointense signal in 6 cases with subdural empyema. Four patients with ventriculitis and six patients with subdural empyema or abscess received surgical drainage, while aggressive treatment was withdrawn in two critically-ill patients upon their parents’ request. Of the eight patients who survived after surgical treatment, three had neurological sequelae at discharge. Subdural effusion was noted in another six patients in whom the brain MRI showed hypointensity on DWI.

The BNCs of seven patients resulted from septic shock and brain hypoperfusion. They had no evidence of bacterial meningitis, but lumbar puncture was performed in only three patients. Their neurologic symptoms included seizure (5), subdural effusion (2), increased intracranial pressure (5), ventriculomegaly (5), and hydrocephalus (4). Only one patient received surgical intervention. An unfavorable outcome was observed in five patients, including death (2), withdraw of life-sustaining treatment (2), and neurological sequelae (1). Only two patients finally survived without neurological sequelae upon discharge.

**Figure 2:**
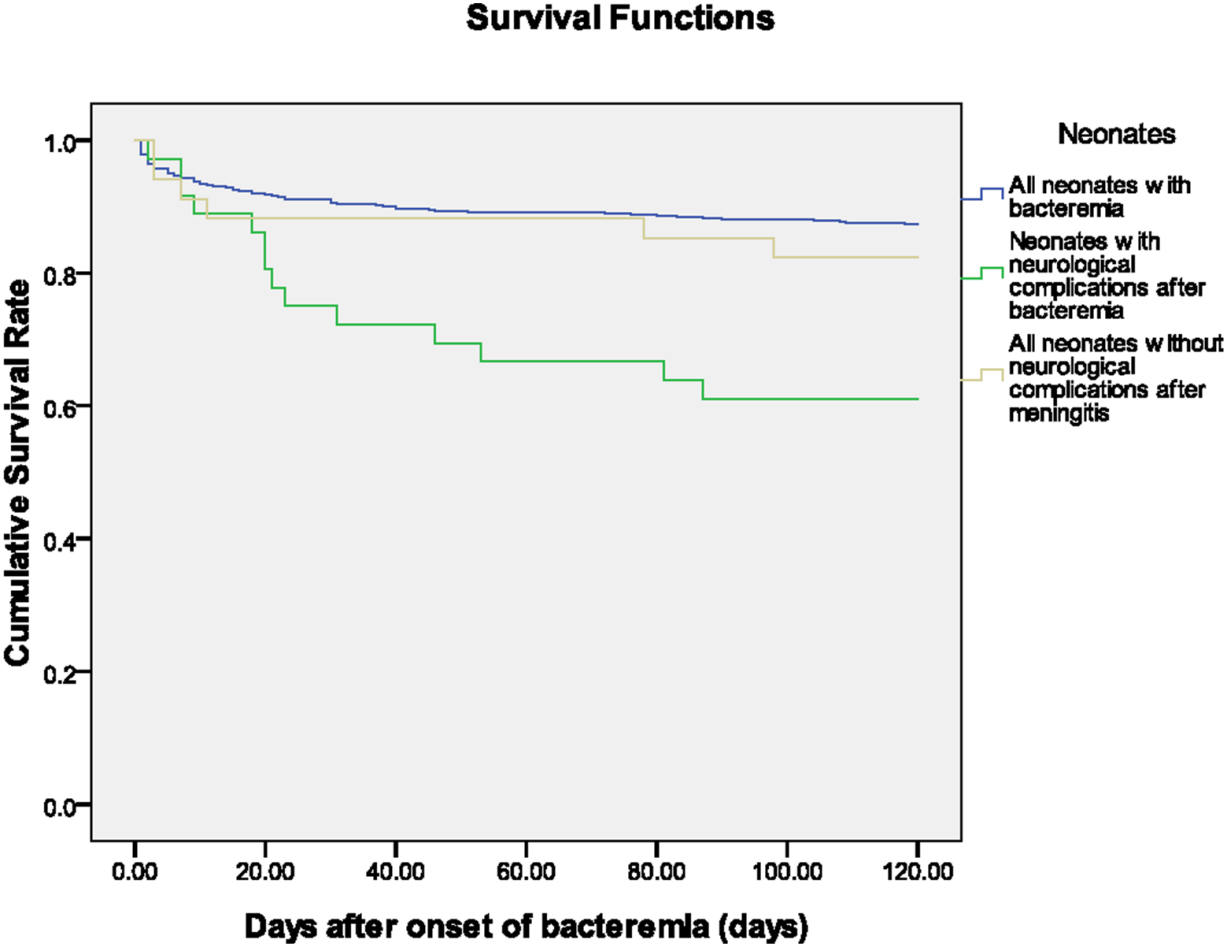
The Kaplan-Meier survival curve of neonates with bacteremia-related neurological complications (BNCs), and those discharged in a critical condition after withdrawing life-sustaining treatments were considered dead at the day of discharge.

Overall, there was a significantly worse outcome in our cohort of neonates with BNCs (both *p*<0.001 by log rank test when compared with neonates without BNCs after bacteremia and meningitis, respectively) (Figure 2). Fourteen (63.6%) of twenty-two survivors who received complete treatment had a favorable outcome without neurological sequelae upon discharge. Eight neonates had motor disabilities at discharge, including opisthotonus posture, drop foot, ankle clonus, and poor feeding and swallowing discoordination. A total of eight patients died, and six patients were discharged after families gave up aggressive treatment and withdrew their life-support. A higher rate of an unfavorable outcome was observed in neonates with acute neurological complications compared to those with meningitis but without acute neurological complications (72% vs. 17%; *p*<0.001).

Because BNCs tended to occur in neonatal bacteremia with concomitant meningitis or septic shock, the relevant subgroup without BNCs constituted the controls. The possible risk factors for BNC are shown in Table 3. By univariate analysis, the risk for BNC varied by gestational age, with full term neonates at greatest risk (OR, 7.08; 95% CI, 2.10–23.90). Pre-existing neurological sequelae were not risk factors for BNC. GBS infection and neonatal meningitis with septic shock increased the likelihood of developing BNCs. After multivariate logistic regression analysis, these two factors remained independently associated with the development of BNCs (adjusted OR: 8.90, 95% CI: 2.20–36.08; *p* = 0.002, and adjusted OR, 5.94; 95% CI: 1.53–23.15, *p* = 0.010, respectively) (Table 3).

**Table 3:** Risk factors for neurological complications in neonatal bacteremia with meningitis or septic shock.

Variable	N (%) (total n = 144)	Unadjusted OR (95% CI)	Multivariate logistic regression analysis	
			Adjusted OR (95% CI)	*P* value
Gestational age				
≤27 weeks	39 (27.1)	1 (ref.)	-	-
28–32 weeks	41 (28.4)	2.12 (0.58–7.71)	-	-
33–36 weeks	26 (18.1)	3.22 (0.84–12.43)	-	-
≥37 weeks	38 (26.4)	7.08 (2.10–23.90)	3.23 (0.81–12.97)	0.098
Male gender	86 (59.7)	0.68 (0.32–1.46)	-	-
Pathogens				
Overall	144 (100)	1 (ref.)		
Group B streptococcus	23 (15.8)	16.13 (4.56–56.99)	8.90 (2.20–36.08)	0.002
Gram-negative bacilli	73 (50.7)	2.41 (0.82–7.10)	2.31 (0.75–7.13)	0.145
Multi-drug resistant bacteria*	10 (6.9)	1.89 (0.71–5.44)	-	-
Pre-existing neurological sequelae	27 (18.8)	0.63 (0.22–1.81)	-	-
Combined meningitis and septic shock	14 (9.7)	10.00 (2.90–34.44)	5.94 (1.53–23.15)	0.010

*Indicating Gram-negative bacilli was resistant to at least three or more of the following antimicrobial categories: carbapenems, penicillins, broad-spectrum cephalosporins, monobactams, aminoglycosides and fluorquinolones.

## Discussion

This study demonstrates that although neurological complications occur only in 3.5% of neonatal bacteremia, they are highly associated with unfavorable outcomes (61.1% had mortality or neurological sequelae). These neurological complications may result from either direct bacterial invasion, sepsis-associated encephalopathy [20], or both. In neonatal bacteremia with concomitant meningitis, 24 (42.1%) cases developed at least one neurological complication in the acute or subacute phase, leading to a complicated clinical course. In neonatal bacteremia with meningitis, GBS and combined septic shock are independently associated with an 8.9- and a 5.9-fold increased risk of developing BNCs. Thus, cranial imaging to detect focal infectious complications or brain pathology is indicated [21].

In contrast to previous studies that have focused on sequelae after childhood meningitis [22], [23], this is the first study investigating neurological complications after bacteremia in the NICU. Meningitis in neonates is associated with a significantly higher rate of acute and subacute neurological complications than in children, which is reported to be around 6.8%–24.0% [22], [23]. Among the BNCs, ventriculitis, subdural empyema and subdural fluid collection were more common in the younger age group based on the current study findings and previous literature [22]–[26]. Mild, transient hydrocephalus and ventriculitis are present in most patients with meningitis [25], [26], but they tend to be more persistent or progressive in young infants with immature neurological development.

Nearly two-thirds of BNCs occur in the late-preterm or full-term infants. Although GBS is well known as the most important pathogen of neonatal early-onset sepsis, 80% (12/15) of our GBS-related BNCs are late-onset sepsis and the majority (13/15, 86.7%) occurred in neonates with a gestational age of ≥33 weeks. A recent prospective report has found that term-born infants have more GBS late-onset sepsis and meningitis compared to preterm infants had [27], even though the incidence is relatively lower in term-born infants (0.24 and 1.4 per 1000 live births for term and preterm newborns, respectively). Given the substantial burden of late-onset GBS disease, an effective prevention strategy should be enforced to reduce maternal colonization and transmission [27], [28].

Based on the time frame of BNC occurrences, nearly half of ventriculomegaly, hydrocephalus, cerebral infarction and encephalomalacia were detected at more than one month after bacteremia or meningitis onset. However, focal infectious complications tended to be detected within half a month after meningitis onset. It can be surmised that these focal infectious complications are the direct extension or persistence of bacterial invasion, and pathological brain would result from subacute neurological damages. The association of these focal infectious complications with an even higher rate of adverse outcome warrants greater awareness. Moreover, some may be delayed diagnosed, because the clinical symptoms are subtle or similar to other BNCs like hydrocephalus or ventriculomegaly. Better strategies aimed at reducing the occurrence of BNCs, such as correct treatment of bacteremia, and more frequent follow-up with neuroimaging studies for early detection of these intracranial lesions are therefore needed.

Risk factors associated with the development of BNCs have not been previously described. Full-term neonates are at an increased risk for BNCs compared to premature infants. However, gestational age-related risks of BNCs are attributable to the high frequency of Group B streptococcus infection in full-term infants. In addition, the presence of pre-existing neurologic sequelae is not independently associated with the development of BNCs, which undoubtedly reduces the possibility of selection bias among the controls. It is reasonable that Group B streptococcus contributes independently to BNCs since Group B streptococcus is the leading cause of meningitis in neonates. Recent studies have found that its special mechanism involves the penetration of the blood-brain barrier [29], [30].

Our study has several important limitations. The clinical features of the patients were retrospectively collected and the sample size was not large enough for a good statistical analysis. It is sometimes difficult to judge whether neonates with pre-existing neurologic sequelae from extreme prematurity had BNCs or progression of neurological deficit after an episode of bacteremia. Furthermore, lack of long-term follow-up for the survivors results in the unavailability of information regarding their neuro-developmental outcomes.

In conclusion, approximately one-fourth of neonates with meningitis or septic shock experience neurologic complications despite prompt instigation of effective antibiotic therapy. Due to the high risk of adverse outcomes after the development of neurologic complications, it is important that physicians can highly alert the possibility of BNCs after bacteremia, especially those caused by Group B streptococcus, and aim to detect neurological complications early at onset of bacteremia to address already present complications and diminish damage and/or progression. Better strategies aimed at reducing the occurrence, aggressive treatment of Group B streptococcus bacteremia, and more frequent cranial ultrasound follow-up are strongly recommended for the screening of infants with bacterial meningitis.
